# P04.49. Herb use among low income women at an urban tertiary care center and their communications with prenatal care providers

**DOI:** 10.1186/1472-6882-12-S1-P319

**Published:** 2012-06-12

**Authors:** K Jarrett, C Pecci, A Filippelli, M Manchu, B Jack, P Gardiner

**Affiliations:** 1 Boston University School of Medicine, Boston, USA; 2 Boston Medical Center, Boston, USA

## Purpose

Little is known about the use of herbs in low income underserved mothers and patterns of communication about herb use to prenatal providers. We sought to examine these issues among women delivering at Boston Medical Center (BMC), an urban medical center serving many of the low income and underserved populations of Boston, Massachusetts.

## Methods

We interviewed women from the inpatient post-natal unit at BMC about what herbs they used during pregnancy, socioeconomic factors, changes to diet, and use of prenatal vitamins. A chart review documented diagnosed medical conditions. We asked women if they discussed the use of herbs with their prenatal care provider, and how satisfied they were with these discussions; we asked the same questions about prenatal vitamin use for comparison.

## Results

Of the 160 women surveyed, 39% reported using herbs and 61% did not. The most commonly used herbs were ginger and peppermint. Herb users were more likely to be high school graduates, identify as black, and have an obstetrical condition. Herb users were more likely to make a dietary change during pregnancy. Only 38% of herb users discussed it with their provider and 82% were satisfied with the conversation. We did not find any medical record documentation of a discussion about herb use. Of the 104 women reporting prenatal vitamin use, 82% discussed it and 91% were satisfied with the conversation.

## Conclusion

In this study 39% of women reported using herbs during pregnancy, a higher frequency than reported in other studies. Few herb users discussed use with their providers, and there was no documentation in the medical record. It is important that providers be given the tools to empower them to discuss herbal medicine use with patients, and are encouraged to do so.

